# Case Report: Primary melanoma of the gastroesophageal junction

**DOI:** 10.12688/f1000research.24302.1

**Published:** 2020-06-02

**Authors:** Yasmine Hussein Agha, Nathaniel A. Parker, Joel Alderson

**Affiliations:** 1 Internal Medicine, University of Kansas School of Medicine, Wichita, KS, 67214, USA; 2 Pathology, Ascension Via Christi St. Francis Hospital, Wichita, KS, 67214, USA

**Keywords:** Esophageal Melanoma, Esophageal Neoplasms, Melanoma, Gastroesophageal junction, Rare Diseases

## Abstract

Primary malignant melanoma represents the fifth most common cancer in the United States. It is subdivided into two forms: cutaneous (90%), visceral (8%, including ocular and mucosal) and of unknown primary (2%). The vast majority of gastrointestinal melanomas are secondary lesions until proven otherwise. Primary esophageal melanoma in particular is exceedingly rare, less than 200 cases have been documented in the literature to date. It is highly prevalent in Japan and occurs twice as much in men than women around the 6th decade of life. It has a predilection for the middle and lower esophagus, with only 6 cases occurring at the gastroesophageal junction worldwide. Its etiology and pathogenesis are poorly understood, and no curative treatment has been established given the paucity of cases. We present a case of primary melanoma of the gastroesophageal junction which represents the 2nd incident case in the united states and 7th worldwide.

## Introduction

Melanoma is the 5 ^th^ most common cancer in the United States and accounts for 5.6% of newly diagnosed cancers ^1^. It is characterized by uncontrolled proliferation of melanocytes which are mainly found in the skin’s epidermis and constitute 91.2% of all melanomas ^2^. Non-cutaneous forms of primary melanoma include ocular and mucosal lesions and represent 5.2% and 1.3% of all melanomas, respectively ^2, 3^. The majority of mucosal subtypes arise in the head and neck and far less commonly in the gastrointestinal and urogenital tracts ^2^. Primary esophageal melanoma (PEM) in particular, is exceedingly rare and accounts for 0.5% of newly identified primary melanomas ^4^. Compared to primary cutaneous melanoma, the risk factors, etiology and pathogenesis of PEM are not well defined; current understanding is derived from cases reported in the literature. We present an unusual case of primary melanoma of the gastroesophageal junction (GEJ).

## Case Description

A 90-year-old Caucasian male presented to emergency department in January 2020 with dysphagia to solid food and 30-pound unintentional weight loss over the past three months. Notable past medical history included gastroesophageal reflux disease, iron deficiency anemia, hypothyroidism, coronary artery bypass grafting, cholecystectomy, and prostate cancer treated with radiation therapy ten years ago. A detailed physical exam of the head, neck, and chest were unremarkable. Vital signs, the remainder of the physical examination, and laboratory testing were unremarkable. Notably, the patient was found to be severely anemic below his baseline blood level with a hemoglobin of 8.1 gm/dL (reference range 12 – 16 gm/dL). Stool guaiac testing was positive. Together with clinical history and laboratory testing an upper gastrointestinal (GI) bleed was suspected.

**Figure 1.**
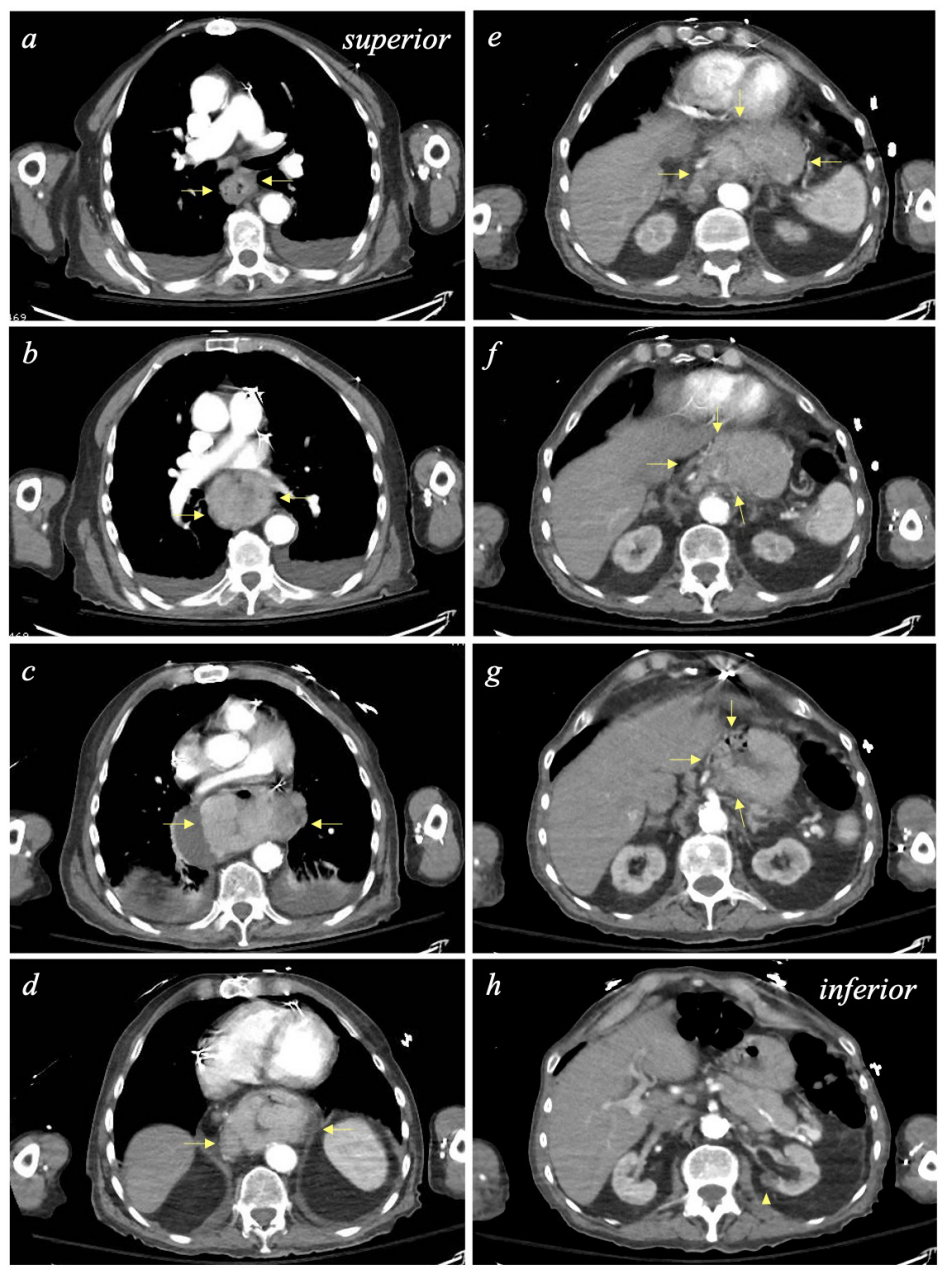
Computed tomography (CT) scans of the chest, abdomen, and pelvis with contrast reveals a large mass. [ *Superior to inferior, alphabetical*] The invasive mass originates in the mid-esophagus ( *top, left*) at the level of the carina and extends into the upper portions of the stomach ( *arrows*). At its center along the gastroesophageal junction (GEJ) the mass reaches its greatest dimensions at 6.1 x 9.36 cm. Enlarged gastrohepatic and paraesophageal lymph nodes are concerning for metastatic disease. ( *Bottom, right*) Left renal lesion with intermediate density ( *arrowhead*).

**Figure 2.**
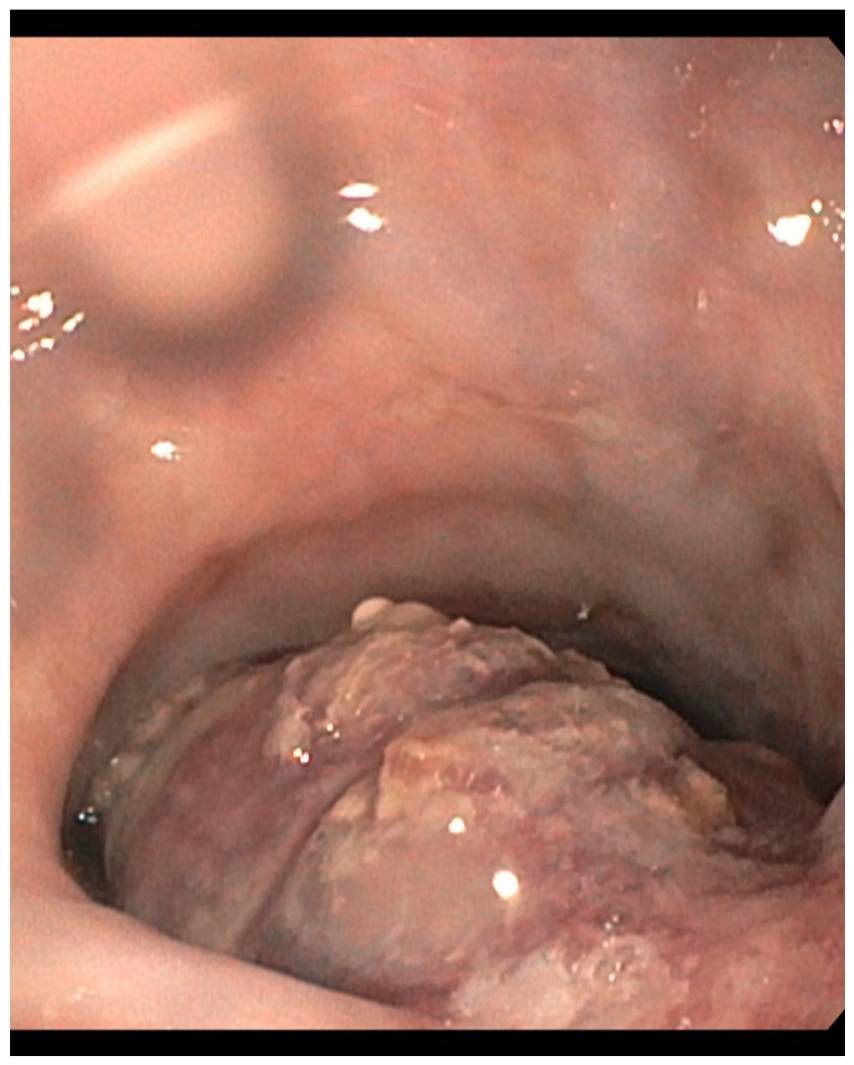
Esophagogastroduodenoscopy (EGD) demonstrates an esophageal mass. The large, necrotic-appearing ulcerated mass is observed to be protruding significantly into the esophageal lumen.

For further workup, computed tomography (CT) scans of the chest, abdomen, and pelvis with contrast were obtained. Imaging revealed a large esophageal mass. Extending approximately 15 cm, the mass appeared to originate in the mid-esophagus and terminate in the upper gastric tissue ( Figure 1). Subsequently, upper GI endoscopy (esophagogastroduodenoscopy; EGD) was performed to determine the underlying cause and exclude malignancy. EGD noted a large, necrotic-appearing ulcerated mass extending 26 cm from the incisors and across the GEJ into the gastric cardia and fundus. Near the GEJ, the mass was obstructive, as it occupied approximately 70% of the esophageal lumen ( Figure 2). Multiple biopsies were obtained along the exposed, protruding portions of the tumor to further evaluate the pathology.

**Figure 3.**
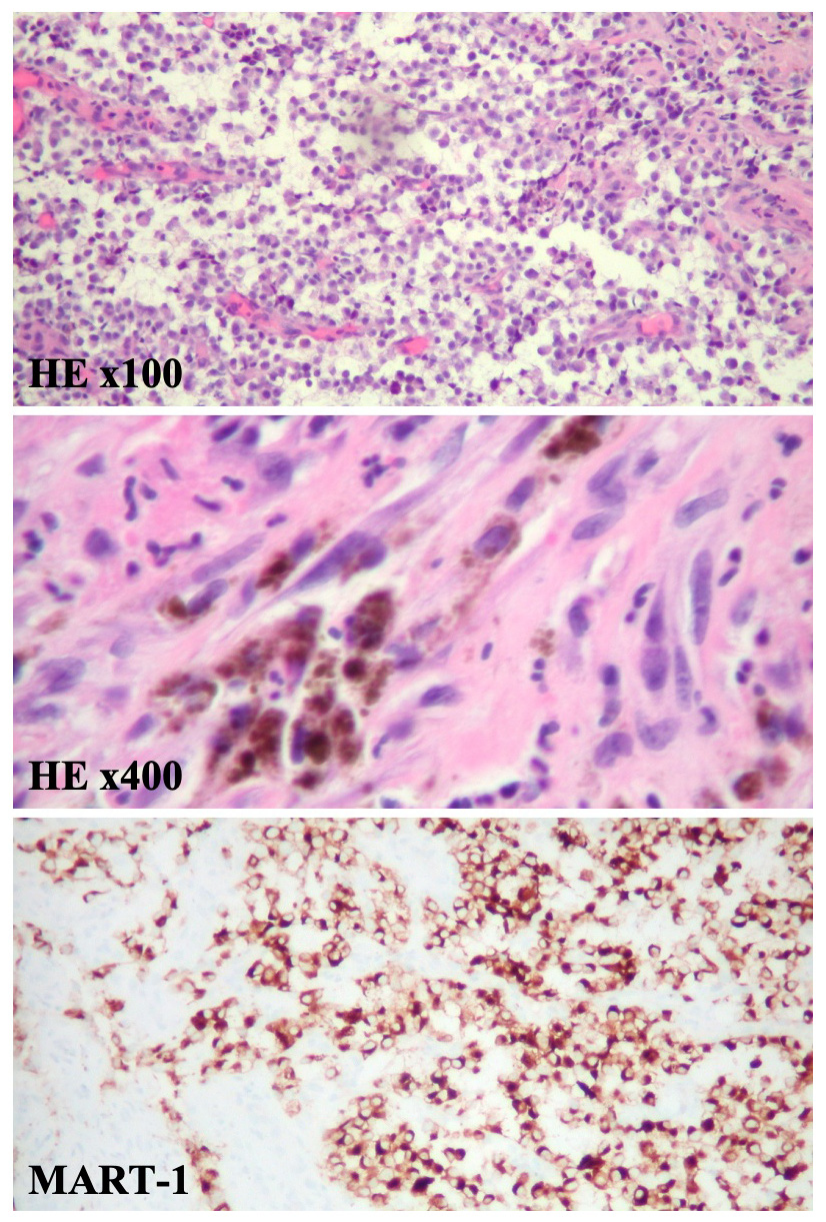
Pathology demonstrates a malignant melanoma. At medium power magnification, histopathology reveals the tumor cells are round with hyperchromatic eccentric nuclei, prominent nucleoli, and mixed eosinophilic and basophilic cytoplasm. Some areas reveal necrosis with neutrophilic exudate. Mitotic activity is frequently seen (HE x100). Pleomorphic melanoma cells containing various amounts of brown melanin pigment (HE x400). At high power magnification, tissue stained with MART-1 (melan-A) by IHC show melanocytes highlighted in brown.

Microscopically, esophageal mass specimens showed sheets of malignant cells. To further classify these tumor cells immunohistochemical (IHC) staining was performed. Tumor cells were positive for MART-1 (melan-A) staining, whereas pankeratin, CD56, synaptophysin, chromogranin, CDX2, p63, and PSA staining was negative ( Figure 3). Based on this IHC profile malignant melanoma was confirmed.

**Figure 4.**
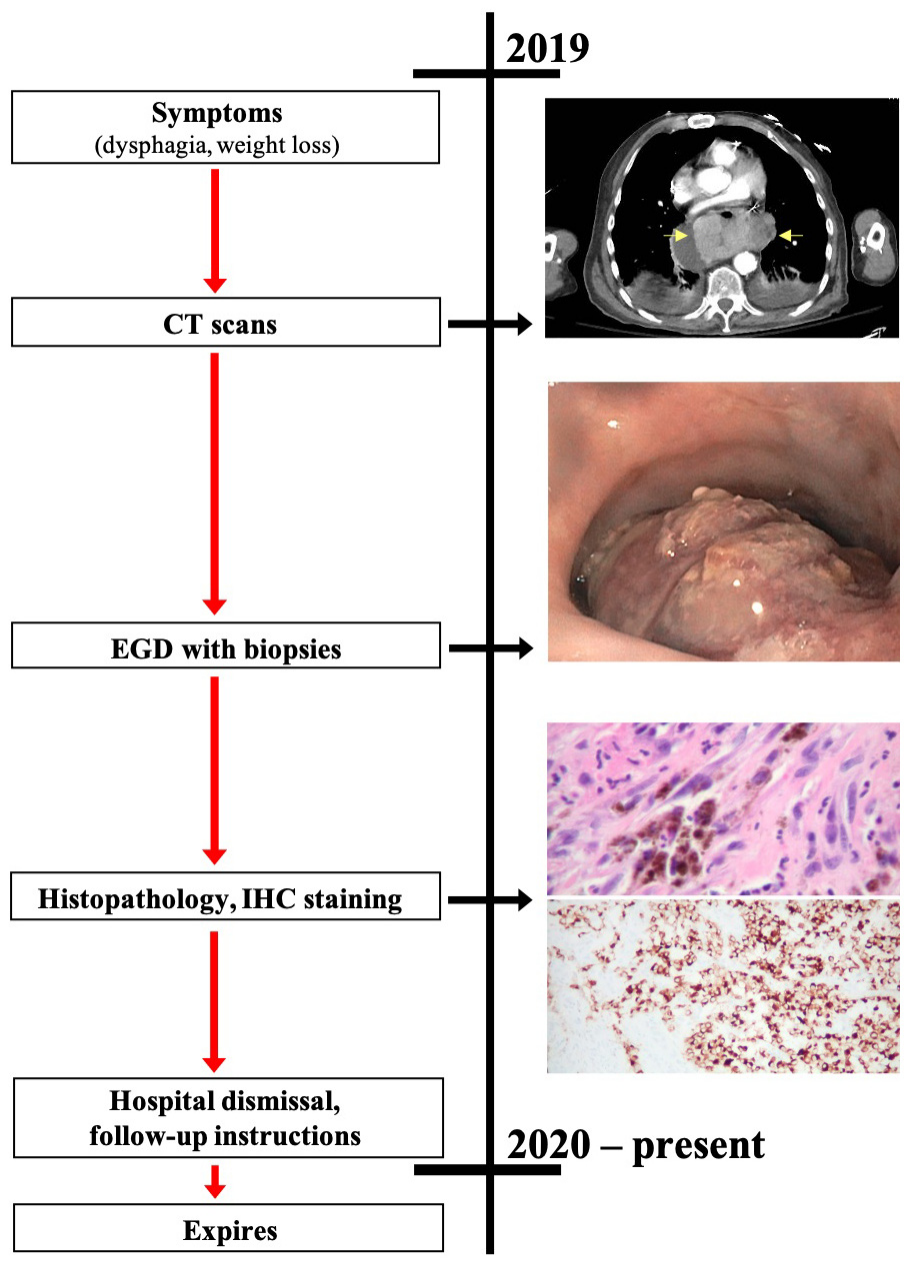
Case report timeline Presented according to CARE guidelines.

 Given the clinical history, presentation, image findings, endoscopic evaluation, and pathology primary malignant melanoma of the GEJ was diagnosed. The patient was dismissed from the hospital with instructions to immediately establish care with his local oncologist. Out-patient orders to check the tumor’s BRAF and programmed death-ligand 1 status, as well as assess for other tumor markers were placed. Two months after hospital dismissal the patient expired ( Figure 4).

## Discussion

Most primary gastrointestinal melanomas have been identified in the oral mucosa and anorectal region. PEM is exceedingly rare; less than 200 cases have been reported in the literature ^5, 6^. It is not only considered a rare subtype of primary melanoma but also accounts for an unusual primary gastrointestinal malignancy as PEM represents 0.1% of all primary esophageal tumors ^4^.

Esophageal melanosis, which is the proliferation of melanocytes and the increase in melanin production within the esophageal mucosa, has been frequently associated with cases describing PEM, however additional risk factors are yet to be defined ^7, 8^. To date, no association between PEM and known carcinogens such as alcohol and tobacco have been recognized in the literature. It occurs more frequently in men than women with an average ratio of 2:1 around the 6 ^th^ decade of life and has a predilection for the Japanese population ^5^. Although melanocytes are mainly skin and choroid bound, Ohashi *et al.* demonstrated in their study that these highly specialized cells can be found in the lower esophagus of up to 8% of healthy Japanese patients ^7^. This correlation could potentially explain the predominance of PEM in this population.

Clinically, patients present with typical symptoms alarming for esophageal malignancy such as dysphagia, sternal chest pain, epigastric pain, hematemesis, melena, decreased appetite and weight loss ^3^. Initial evaluation should include upper endoscopy and biopsy followed by review of the tissue’s pathology and immunohistochemical staining to make a diagnosis ^9^. These tumors are generally located in the middle and lower esophageal lumen, are polypoid and can measure up to 5 cm ^5, 10^. 70% of these tumors are non-ulcerated and pigmented, while the rest can be amelanotic and necrotic ^4, 5^. S-100 is a highly sensitive marker for melanoma; HBM-45, melan-A, MITF and tyrosinase are highly specific markers for this entity ^11^. Finally, although most PEM are non-metastatic at presentation, re-assessing the patient with a thorough physical exam and full body positron emission tomography / CT is necessary to rule out other primary tumors and to stage the current disease.

Given the paucity of cases, no general consensus exists regarding the optimal management of PEM. Schizas *et al.* analyzed in their study the outcomes following treatment of 93 individuals with PEM. Most patients underwent esophagectomy and patients who received adjuvant chemotherapy or immunotherapy had reduced rates of relapse; however overall survival (OS) was unchanged compared to those who had surgery only ^5, 12^. The choice of systemic therapy was derived from the guidelines to treat primary malignant melanoma during the time the patient was diagnosed. These drugs included cisplatin, vincristine, and dacarbazine ^4, 13^. OS rates for all patients regardless of whether they underwent radical resection only or had adjuvant therapy was 4.5% ^5^. Three additional retrospective studies also showed poor prognosis regardless of management with median OS of 10 and 18 months ^4, 13, 14^.

To the best of our knowledge, this is the 7 ^th^ case of PEM arising at the gastroesophageal junction worldwide and second case documented in the United States ^15– 20^. Timely diagnosis and work-up of this patient represent major strengths in managing this case to avoid delay in treatment and worse outcomes. The lack of guidelines for management of patients with PEM however are a major limitation and his unfortunate early death did not allow us to initiate treatment and evaluate his clinical response in an attempt to set ground for management of future patients diagnosed with PEM.

## Conclusion

PEM is an aggressive and uncommon cancer with less than 200 cases worldwide. Its incidence is highest among the Japanese population and could possibly be correlated with the increased prevalence of esophageal melanosis in this group. It occurs around the age of 60 years and has a predilection for men. The etiology, risk factors and tumorigenesis are poorly understood, and no general consensus exists regarding an optimal management for this cancer. Current practices include esophagectomy with adjuvant chemotherapy or immunotherapy and is based on guidelines to treat primary cutaneous melanoma. Prognosis is poor, median survival is less than one year.

## Consent

Written informed consent for publication of clinical details and clinical images was obtained from the patient himself prior to his death.

## Data availability

### Underlying data

All data underlying the results are available as part of the article and no additional source data are required.
