# Supplementary material for: Case Report: Primary melanoma of the gastroesophageal junction
Source: F1000Res. 2020 Jun 2;9:490. [Version 1] doi: 10.12688/f1000research.24302.1 (PMC7338914; doi:10.12688/f1000research.24302.1)
Supplement: Peer review history

# Peer review: Reviewer response for version 1

**DOI:** 10.5256/f1000research.26812.r65044

**Published:** 2020-07-06

**Authors:** Gregory W. Roloff

**Affiliations:** 1 Loyola Medicine (Loyola University Health System), Chicago, IL, USA

The report by Agha and colleagues is well-researched and well-written, describing an uncommon presentation of a GE junction melanoma. The time course is clearly delineated and the timeline figure allows the reader a full appreciation for the significance of the patient's unfortunate but not unexpected mortality given the debilitation associated with the anatomic location of the lesion. In order to more clearly describe the tumor, it would be helpful if the grade and stage could be included. Namely, was PET-CT conducted to establish distant metastasis? Otherwise, the case is strong and will serve as a helpful addition to the literature.

Are enough details provided of any physical examination and diagnostic tests, treatment given and outcomes?

Yes

Is the case presented with sufficient detail to be useful for other practitioners?

Yes

Is sufficient discussion included of the importance of the findings and their relevance to future understanding of disease processes, diagnosis or treatment?

Yes

Is the background of the case’s history and progression described in sufficient detail?

Yes

Reviewer Expertise:

Internal Medicine, hematology/oncology

I confirm that I have read this submission and believe that I have an appropriate level of expertise to confirm that it is of an acceptable scientific standard.

# Peer review: Reviewer response for version 1

**DOI:** 10.5256/f1000research.26812.r64198

**Published:** 2020-06-05

**Authors:** Mahmoud Al-Rifai

**Affiliations:** 1 Section of Cardiology, Baylor College of Medicine, Houston, TX, USA

Very interesting and rare case presentation of gastrointestinal melanoma occurring at the GE junction. A sufficient background and case presentation were presented followed by a well thought of discussion. This case is not only novel but also highly important to describe in the literature given its rarity and lack of proper understanding of risk factors, tumorigenesis, and ultimately treatment. Unfortunately the patient expired which is not surprising given poor prognosis of this disease.  

 I would suggest the authors include grade and stage of the tumor. It appears that the tumor was not metastatic on presentation but this needs to be clearly established.

Are enough details provided of any physical examination and diagnostic tests, treatment given and outcomes?

Yes

Is the case presented with sufficient detail to be useful for other practitioners?

Yes

Is sufficient discussion included of the importance of the findings and their relevance to future understanding of disease processes, diagnosis or treatment?

Yes

Is the background of the case’s history and progression described in sufficient detail?

Yes

Reviewer Expertise:

Medicine, CVD

I confirm that I have read this submission and believe that I have an appropriate level of expertise to confirm that it is of an acceptable scientific standard.
